# Singular versus combinatory glucose-sensitive signal control of metabolic sensor protein profiles in hypothalamic astrocyte cultures from each sex

**DOI:** 10.1515/tnsci-2022-0259

**Published:** 2022-11-30

**Authors:** Abdulrahman Alhamyani, Prabhat R. Napit, Khaggeswar Bheemanapally, Paul W. Sylvester, Karen P. Briski

**Affiliations:** School of Basic Pharmaceutical and Toxicological Sciences, College of Pharmacy, University of Louisiana Monroe, Rm 356 Bienville Building, 1800 Bienville Drive, Monroe, LA 71201, United States; Pharmaceuticals Chemistry Department, Faculty of Clinical Pharmacy, Al Baha University, Al Baha city, 65779, Saudi Arabia

**Keywords:** dexamethasone, norepinephrine, GLUT2, glucokinase, AMPK, glucocorticoid receptor

## Abstract

Brain metabolic-sensory targets for modulatory glucose-sensitive endocrine and neurochemical signals remain unidentified. A hypothalamic astrocyte primary culture model was here used to investigate whether glucocorticoid receptor (GR) and noradrenergic signals regulate astrocyte glucose (glucose transporter-2 [GLUT2], glucokinase) and/or energy (5′-AMP-activated protein kinase [AMPK]) sensor reactivity to glucoprivation by sex. Glucose-supplied astrocytes of each sex showed increased GLUT2 expression after incubation with the GR agonist dexamethasone (DEX) or norepinephrine (NE); DEX plus NE (DEX/NE) augmented GLUT2 in the female, but not in male. Glucoprivation did not alter GLUT2 expression, but eliminated NE regulation of this protein in both sexes. Male and female astrocyte glucokinase profiles were refractory to all drug treatments, but were down-regulated by glucoprivation. Glucoprivation altered AMPK expression in male only, and caused divergent sex-specific changes in activated, i.e., phosphoAMPK (pAMPK) levels. DEX or DEX/NE inhibited (male) or stimulated (female) AMPK and pAMPK proteins in both glucose-supplied and -deprived astrocytes. In male, NE coincidently up-regulated AMPK and inhibited pAMPK profiles in glucose-supplied astrocytes; these effects were abolished by glucoprivation. In female, AMPK profiles were unaffected by NE irrespective of glucose status, whereas pAMPK expression was up-regulated by NE only during glucoprivation. Present outcomes document, for each sex, effects of glucose status on hypothalamic astrocyte glucokinase, AMPK, and pAMPK protein expression and on noradrenergic control of these profiles. Data also show that DEX and NE regulation of GLUT2 is sex-monomorphic, but both stimuli impose divergent sex-specific effects on AMPK and pAMPK. Further effort is warranted to characterize mechanisms responsible for sex-dimorphic GR and noradrenergic governance of hypothalamic astrocyte energy sensory function.

## Abbreviations

AMPK: 5′-AMP-activated protein kinase
CaMKKβ: calcium/calmodulin-dependent protein kinase kinase-beta
DEX: dexamethasone
GCK: glucokinase
GLUT2: glucose transporter-2
IIH: insulin-induced hypoglycemia
NE: norepinephrine
pAMPK: phosphoAMPK
PP1: protein phosphorylase-1

## 1 Introduction

The glucose-regulatory neural circuitry monitors cellular metabolic status and systemic energy substrate storage to exert appropriate control of central and peripheral effector motor functions [1–5]. Many metabolic sensory mechanisms are utilized in the brain, including screening of the critical nutrient glucose or the energy currency ATP. Glucose is monitored at the point of entry into the cell by the low affinity, high Km glucose transporter-2 (GLUT2) [6], and also at the rate-limiting step of glycolysis by the specialized hexokinase glucokinase (GCK) [7]. Deviations in cellular ATP levels are communicated by the ultra-sensitive energy sensor 5′-AMP-activated protein kinase (AMPK), which is activated in response to decrements in the AMP/ATP ratio. The hypothalamus, the principal autonomic motor center in the brain, exerts final control over autonomic, neuroendocrine, and behavioral outflow that rectifies glucose deficiency [8]. Hypothalamic GLUT2, GCK, and AMPK provide critical input to neural pathways that govern body-wide energy and glucose stability [9–18]. The identity(-ies) of hypothalamic cell types that express these sensors remains unclear.

Brain neuroglia are key participants in neuro-metabolic homeostasis [19–22]. Astrocytes support optimal nerve cell function by multiple mechanisms, including provision of metabolic fuel [23]. Glucose, the principal energy source to the brain, is taken up from the circulation by astrocytes, and is either stored in those cells by incorporation into the complex polymer glycogen or is catabolized via the glycolytic pathway to the oxidizable fuel l-lactate for trafficking to neurons [24]. *In vivo* studies using combinatory immunocytochemistry, laser-catapult microdissection, and Western blotting show that astrocytes from the female rat express hypoglycemia-sensitive AMPK [25]. It is intriguing to consider whether astrocytes engage in glucose sensing, and if so, how monitored decrements in glucose utilization correlate with AMPK activity state in each sex. Current research used a characterized hypothalamic primary astrocyte culture model (Ibrahim et al., [26–28]) to investigate the premise that hypothalamic astrocytes from male and female rats express GLUT2 and GCK as well as AMPK, and that glucose and energy sensor protein profiles and/or AMPK activation status may exhibit sex-dimorphic responses to glucoprivation.

The hypothalamus is a principal target for modulatory actions of glucose-sensitive endocrine and neurochemical stimuli. The hypothalamic–pituitary–adrenal neuroendocrine axis is activated by various stressors, including hypoglycemia. Adrenoglucocorticoid hormones act via classical/type II glucocorticoid receptors (GRs) expressed in the brain and other organs to regulate systemic glucose homeostasis through control of glucose production, uptake, and storage [29,30]. While glucocorticoid-sensitive astrocytes were originally identified in the cerebral cortex and cerebellum [31], recent studies show that hypothalamic astrocytes also express GRs (Briski, personal communication). Central aminergic autonomic pathways are also activated in reaction to hypoglycemia, resulting in augmented noradrenergic signaling to key forebrain structures including the hypothalamus [32]. Norepinephrine (NE) is a vital glucose-regulatory stimulus that links hindbrain dorsal vagal complex metabolic-sensory A2 noradrenergic neurons with hypothalamic metabolic loci [33]. Hypothalamic astrocytes are direct targets for NE actions as these cells express alpha_1_-adrenergic receptor (α1-AR), alpha_2_-AR (α2-AR), and beta_1_-AR (β1-AR) proteins [26,34]. The possibility that GR or noradrenergic stimuli may modulate hypothalamic astrocyte metabolic sensor reactivity to glucoprivation has not been investigated. A corollary objective of the present project was to address the hypothesis that GRs and NE impose singular and/or interactive control of glucose and energy sensor protein and AMPK activation during glucoprivation, and that the direction and/or magnitude of such control may vary according to sex.

## 2 Materials and methods

### 2.1 Primary astrocyte cell cultures

High-purity hypothalamic astrocyte primary cultures were prepared from adult (2–3 months of age) male or female rat brains, as previously described [26–28].

**Figure 1:**
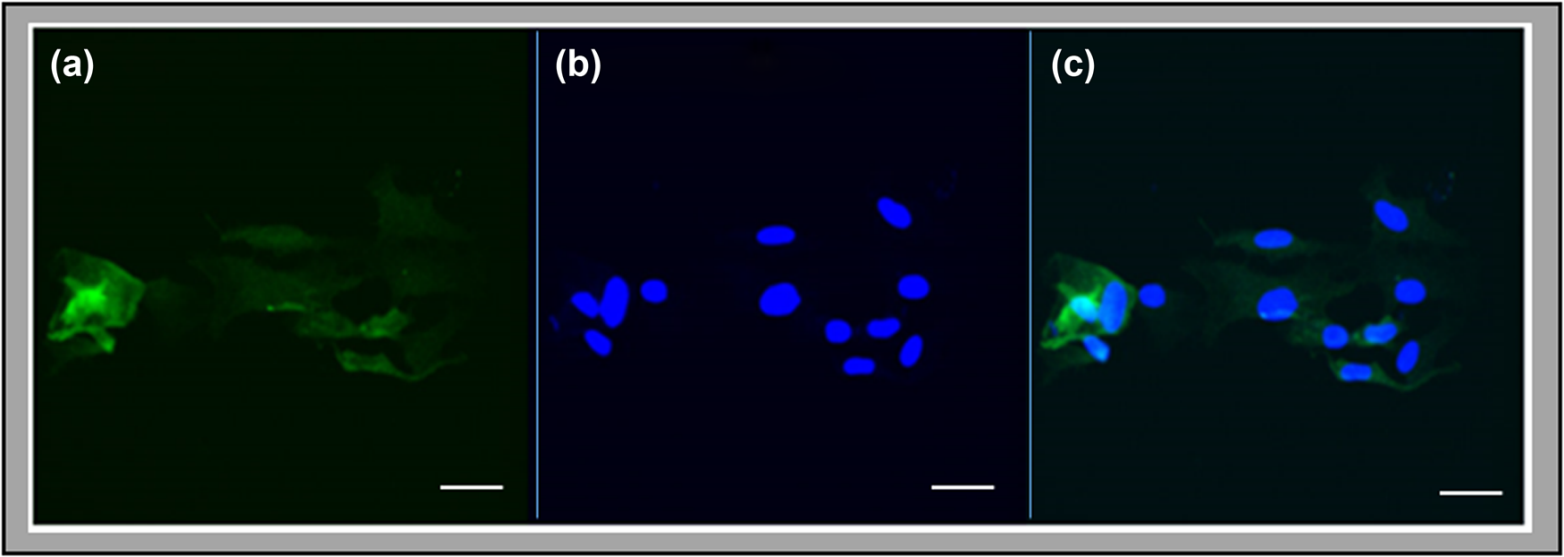
Immunofluorescence staining of hypothalamic primary astrocyte cell cultures for the astrocyte-specific marker GFAP. Cultured astrocytes were immunostained for cytoplasmic GFAP immunoreactivity (left-hand panel (a) green fluorescence) and exposed to the DNA stain DAPI (middle panel (b) blue fluorescence), and imaged with a Zeiss LSM 5 PASCAL confocal scanning laser microscope. The right-hand panel in (c) shows a merged image of these staining patterns. Scale bar = 50 μm.

Briefly, dissected whole-hypothalamus tissue blocks were digested with 2.5% trypsin (prod. no. 15090-046; Thermo Fisher Scientific, Waltham, MA), then dissociated by pipet into a single-cell suspension in DMEM high-glucose media (prod. no. 12800-017; Thermo Fisher Scientific, Waltham, MA) supplemented with 10.0% heat-inactivated fetal bovine serum (FBS; GE Healthcare Bio-Sciences, Pittsburgh, PA) and 1.0% penicillin/streptomycin (prod. no. 15140-122; Thermo Fisher Scientific). Dissociated cells were incubated (50 μg/mL initial concentration) in poly-d-lysine (prod. no. A-003-E; MilliporeSigma, Burlington, MA)-coated T75 culture flasks for 2 weeks prior to removal of microglia and oligodendrocytes. Approximately 7–8 days after plating, microglia were removed from cultures by aspiration after shaking at 180 rpm (30 min). Cells were re-suspended in fresh media (20 mL) and shaken first on an orbital platform at 240 rpm (6–8 h), then vigorously by hand (1 min) before aspiration to discard oligodendrocyte precursor cells, as described [26]. Purified astrocytes were then incubated for 12–14 days with media changes at 2–3 day intervals. Routine Western blot and immunofluorescence cytochemical detection of the astrocyte marker protein glial fibrillary acidic protein (GFAP) was performed to verify astrocyte culture purity [26–28]. Astrocytes were plated at 60–80% approximate density and allowed to attach to poly-d-lysine-coated 8-chamber slides 1 day prior to staining. Cells were washed with phosphate-buffered saline, fixed with 4% paraformaldehyde, then permeabilized with 0.1% Triton X-100 (TX) (prod. no. T9284; Sigma-Aldrich, St. Louis, MO). After blocking with 10% normal goat serum (NGS) (prod. no. S-1000; Vector Laboratories, Burlingame, CA, USA), cells were incubated with a mouse antiserum against GFAP (1:250; prod. no. 3670S; Cell Signaling Technol., Danvers, MA) for 48 h at 4°C, then exposed (1 h; room temperature; under dark) to goat-anti-mouse FITC-conjugated secondary antibodies (1:2,000; prod. no. A10530; Thermo Fisher Scientific), diluted in 10% NGS containing TX. DNA staining was accomplished using 4′,6-diamidino-2-phenylindole (DAPI). Cells were cover slipped with Vectashield fluorescence mounting medium (prod. no. H-1000; Vector Lab.). GFAP immunoreactive (-ir)- and DAPI-epifluorescence positive astrocytes were imaged with a Zeiss LSM 5 PASCAL confocal scanning laser microscope (Carl Zeiss Micro-Imaging Inc., Thornwood, NY, USA). Images in Figure 1 show that culture purity exceeded 95%.

### 2.2 Experimental design

Astrocytes were plated at a density of 1 × 10^6^ cells/100 mm^2^ in poly-d-lysine-coated culture and maintained in DMEM high-glucose media. Cultures attaining approximately 70% confluency were incubated for 18 h in DMEM high-glucose media containing 5.0% charcoal-stripped FBS (prod. no. 12676029; Thermo Fisher Scientific). Astrocytes from each sex were next incubated for 8 h in 10 nM 17β-estradiol-supplemented HBSS media containing 5.5 mM glucose (G_5.5_) or 0 mM glucose (G_0_) [28]. Glucose-supplied and glucose-deprived cultures were treated with NE (10 nM [26]; prod. no. A7257; Sigma-Aldrich, St. Louis, MO), dexamethasone (DEX) (100 nM [35]; prod. no. D4902; Sigma-Aldrich), or NE (10 nM) plus DEX (100 nM) dissolved in dimethyl sulfoxide (DMSO). DMSO was administered in an equal volume to all treatment groups to achieve a uniform media concentration of 0.01%. Controls were incubated with HBSS media containing DMSO alone. Astrocytes were detached from plates with 0.05% trypsin EDTA (prod. no. 25300-062; Thermo Fisher Scientific), washed in PBS, pelleted in lysis buffer (2.0% sodium dodecyl sulfate, 10.0% glycerol, 5% β-mercaptoethanol, 1 mM sodium orthovanadate [protease inhibitor], 60 mM Tris–HCl, pH 6.8), heat-denatured (10 min, 95°C), or ultra-pure water for Western blot protein or LC-ESI-MS glycogen analyses, respectively.

### 2.3 Western blot analysis

Astrocyte cell pellets were heat-denatured (10 min, 95^o^C), sonicated, centrifuged, and diluted with 2× Laemmli buffer. Sample protein concentrations were determined by NanoDrop spectrophotometry (prod. no. ND-ONE-W; Thermo Fisher Scientific). For each protein of interest, pooled sample aliquots of equivalent protein mass were loaded for each treatment group for separation in Bio-Rad Stain Free 10% acrylamide gels (prod. no 161-0183; Bio-Rad, Hercules, CA), as described [36,37]. Astrocyte target proteins were analyzed in three independent experiments. After UV gel activation (1 min) in a Bio-Rad ChemiDoc™ Touch Imaging System, proteins were transblotted to 0.45-µm PVDF-Plus membranes (prod. no. 1212639; Data Support Co., Panorama City, CA). Membrane buffer washes and antibody incubations were performed by Freedom Rocker™ Blotbot^®^ automation (Next Advance, Inc., Troy, NY). After blocking (2 h) with Tris-buffered saline, pH 7.4, containing 0.2% Tween-20 (prod. no. 9005-64-5; VWR, Radnor, PA) and 2% bovine serum albumin (prod. no. 9048-46-8; VWR), membranes were incubated (24–48 h; 4^o^C) with primary antisera raised in rabbit against GLUT2 (1:1,000; prod. no. PA5-97263; Thermo Fisher Scientific), GCK (1:1,000; prod. no. BS-1796R; Bioss Antibodies, Woburn, MA), AMPK_α1/2_ (1:2,000; prod. no.2532S; Cell Signaling Technol., Danvers, MA), phosphoAMPK_α1/2_-Thr 172 (1:1,600; prod. no. 2535S; Cell Signaling Technol.), the upstream stimulatory kinase calcium/calmodulin-dependent protein kinase kinase-beta (CaMKKβ; 1:1,000; prod. no. 16810S; Cell Signaling Technol.), the upstream inhibitory phosphatase protein phosphatase-1 (PP1; 1:1,000; prod. no. 2582S; Cell Signaling Technol.), α1AR (1:1,000; prod. no. PA1-047; Thermo Fisher Scientific), α2AR (1:1,000; prod. no. PA1-048; Thermo Fisher Scientific), or β1AR (1:1,000; prod. no. NBP1-59007; Novus Biologicals, Littleton, CO), or GR (1:1,000; prod. no. 3660S; Cell Signaling Technol.). Membranes were next incubated (1 h) with goat anti-rabbit horseradish peroxidase-conjugated secondary antibodies (prod. no. NEF812001EA; 1:4,000; PerkinElmer, Waltham, MA), followed by Supersignal WestFemto maximum sensitivity chemiluminescent substrate (prod. no. 34096; Thermo Fisher Scientific). Target protein optical density (O.D.) signals were quantified in the ChemiDoc™ Touch Imaging System described above, and normalized to total in-lane protein, e.g., all protein electrophoresed in the individual sample lane, using Bio-Rad proprietary stain-free imaging gel technology and Image Lab™ 6.0.0 software (http://www.bio-rad.com/en-us/applications-technologies/stain-free-imaging-technology?ID=NZ0G1815), as reported [26,28]. This superior method for Western blot normalization markedly reduces data variability through improved measurement accuracy and precision [38,39]. Precision plus protein molecular weight dual color standards (prod. no. 161-0374; Bio-Rad) were included in each Western blot analysis in the current study.

### 2.4 Statistics

Mean normalized protein O.D. were evaluated between treatment groups within each sex by two-way analysis of variance and Student Newman Keuls *post-hoc* test. Differences of *p* < 0.05 were considered significant. In each figure, statistical differences between specific pairs of treatment groups are denoted as follows: *p < 0.05; **p < 0.01; and ***p < 0.001.


**Ethical approval:** The research related to animals’ use has been complied with all the relevant national regulations and institutional policies for the care and use of animals. All animal protocols were performed in compliance with the National Institutes of Health Guide for the Care and Use of Laboratory Animals, 8th Edition, under University of Louisiana at Monroe Institutional Animal Care and Use Committee approval. Sex of animals used is included, along with discussion of sex impacts on study outcomes.

## 3 Results

**Figure 2:**
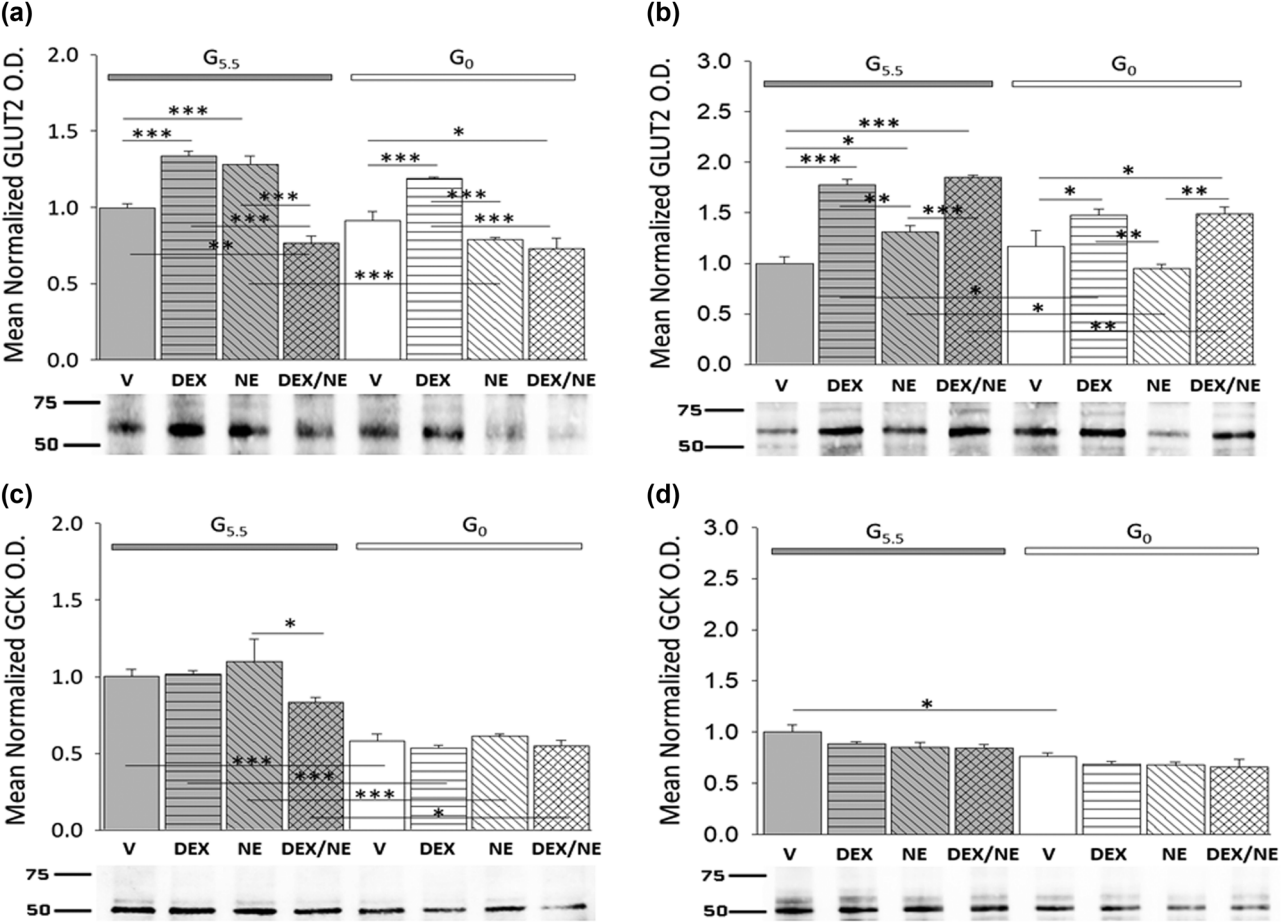
Effects of the GR agonist DEX alone or in combination with the catecholamine transmitter NE on GLUT2 and GCK protein expression in glucose-supplied or glucose-deprived male versus female hypothalamic primary astrocyte cultures. Astrocyte cultures derived from adult rats of each sex were pre-incubated in steroid-free media (18 h) prior to incubation (8 h) with HBSS media supplemented with 5.5 (G5.5) or 0 mM (G0) glucose containing DEX (10 nM), NE (100 nM), DEX (10 nM) plus NE (100 nM), or vehicle (V; DMSO) alone. Data show mean normalized GLUT2 (a: male *n* = 3 independent experiments per treatment group and b: female *n* = 3 independent experiments per treatment group) or GCK (c: male *n* = 3 independent experiments per treatment group and d: female *n* = 3 independent experiments per treatment group) protein O.D. measures ± S.E.M. for male and female G5.5- (gray bars) or G0- (white bars) exposed astrocytes divided into the following treatment groups: V (solid bars), DEX (horizontal-striped bars), NE (diagonal-striped bars), DEX/NE (cross-hatched bars). Data were analyzed by two-way ANOVA and Student Newman Keuls *post-hoc* test. Statistical differences between treatment groups are indicated by the following symbols: *p < 0.05; **p < 0.01; and ***p < 0.001.

Research outcomes described below were obtained using a characterized hypothalamic primary astrocyte culture model to address the question of whether hypothalamic astrocyte primary cultures established from male and/or female rats express glucose-sensitive plasma membrane and glycolytic pathway glucose sensors, and these nutrient gauges are regulated by glucocorticoid or noradrenergic input alone or by interaction of these regulatory stimuli. Figure 2 depicts singular or combinatory effects of the GR agonist DEX and AR ligand NE on hypothalamic primary astrocyte GLUT2 (Figure 2a [male; *F*_(7,16)_ = 32.40, *p* < 0.001; glucose status main effect: *F*_(1,16)_ = 39.83, *p* < 0.001; treatment main effect: *F*_(3,16)_ = 50.43, *p* < 0.001; glucose status/treatment interaction: *F*_(3,16)_ = 11.90, *p* < 0.001] and Figure 2b [female; *F*_(7,16)_ = 19.86, *p* < 0.001; glucose status main effect: *F*_(1,16)_ = 16.63, *p* = 0.001; treatment main effect: *F*_(3,16)_ = 34.84, *p* < 0.001; glucose status/treatment interaction: *F*_(3,16)_ = 5.97, *p* = 0.006]) and GCK (Figure 2c [male; *F*_(7,16)_ = 15.22, *p* < 0.001; glucose status main effect: *F*_(1,16)_ = 95.43, *p* < 0.001; treatment main effect: *F*_(3,16)_ = 2.49, *p* = 0.097; glucose status/treatment interaction: *F*_(3,16)_ = 1.20, *p* = 0.342] and Figure 2d [female; *F*_(7,16)_ = 6.70, *p* = 0.001; glucose status main effect: *F*_(1,16)_ = 36.47, *p* < 0.001; treatment main effect: *F*_(3,16)_ = 3.25, *p* = 0.050; glucose status/treatment interaction: *F*_(3,16)_ = 0.220, *p* = 0.881]) protein expression in each sex. For each sex, treatment groups including astrocytes supplied with 5.5 mM glucose (G_5.5_), *at left*, are illustrated by gray bars, while glucose-deprived (G_0_) treatment groups, *at right*, are shown in white. Data indicate that DEX or NE alone each significantly increased GLUT2 levels in glucose-supplied male and female astrocytes (G5.5/DEX [horizontal-striped gray bars] or G5.5/NE [diagonal-striped gray bars] versus G5.5/V [solid gray bars]). DEX and NE co-incubation (cross-hatched gray bars) reduced (male) or elevated (female) GLUT2 content relative to vehicle controls. In each sex, GLUT2 protein profiles were unaffected by glucoprivation (G0/V [solid white bars] versus G5.5/V). In glucose-deprived male and female astrocytes, GLUT2 profiles were elevated by DEX (G0/DEX [horizontal-striped white bars] versus G0/V), but were refractory to NE (G0/NE [diagonal-striped white bars] versus G0/V); DEX plus NE treatment (cross-hatched white bars) increased GLUT2 expression to levels measured after incubation with DEX alone. In each sex, hypothalamic astrocyte GCK content was decreased in response to glucoprivation, but was altered by any drug treatment when glucose was present or absent.

**Figure 3:**
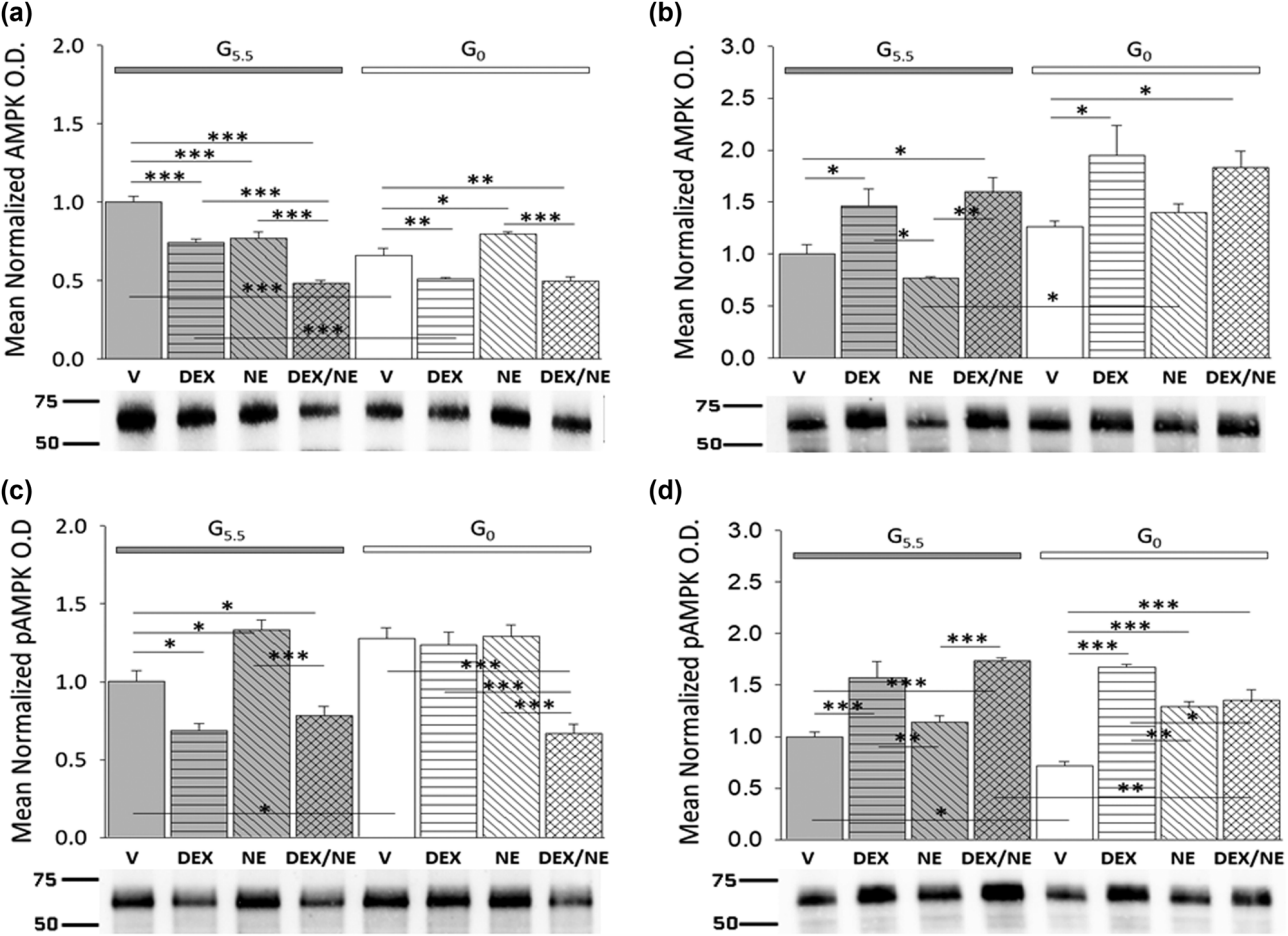
Impact of glucose provision versus starvation on DEX- or NE-associated patterns of AMPK and pAMPK protein expression in male and female hypothalamic primary astrocyte cultures. Data illustrate mean normalized AMPK or pAMPK protein O.D. values ± S.E.M. for glucose-suppled (G5.5; gray bars) or glucose-deprived (G0; white bars) male (a and c: *n* = 3 independent experiments per treatment group) and female (b and d: *n* = 3 independent experiments per treatment group) astrocytes assigned to the following treatment groups: G5.5- or G0-V (solid bars), G5.5- or G0-DEX (horizontal-striped bars), G5.5- or G0-NE (diagonal-striped bars), G5.5- or G0-DEX/NE (cross-hatched bars). Data were analyzed by two-way ANOVA and Student Newman Keuls *post-hoc* test. Statistical differences between treatment groups are indicated by the following symbols: *p < 0.05; **p < 0.01; and ***p < 0.001.

The ultra-sensitive energy gauge AMPK monitors the cellular AMP/ATP ratio. Current work examined the premise that hypothalamic astrocyte total AMPK protein and/or activated, e.g., phosphorylated AMPK (pAMPK) protein profiles are affected by glucose withdrawal in a sex-specific manner, and the related question that AMPK activation may be differentially controlled by DEX or NE when glucose is present versus absent. Data presented in Figure 3 illustrate DEX and/or NE regulation of male or female hypothalamic astrocyte AMPK (Figure 3a [male; *F*_(7,16)_ = 37.09, *p* < 0.001; glucose status main effect: *F*_(1,16)_ = 38.86, *p* < 0.001; treatment main effect: *F*_(3,16)_ = 54.84, *p* < 0.001; glucose status/treatment interaction: *F*_(3,16)_ = 18.78, *p* < 0.001] and Figure 3b [female; *F*_(7,16)_ = 7.32, *p* = 0.001; glucose status main effect: *F*_(1,16)_ = 15.07, *p* = 0.001; treatment main effect: *F*_(3,16)_ = 11.24, *p* < 0.001; glucose status/treatment interaction: *F*_(3,16)_ = 0.82, *p* = 0.499]) and pAMPK (Figure 3c [male; *F*_(7,16)_ = 19.16, *p* < 0.001; glucose status main effect: *F*_(1,16)_ = 13.28, *p* = 0.002; treatment main effect: *F*_(3,16)_ = 29.35, *p* < 0.001; glucose status/treatment interaction: *F*_(3,16)_ = 10.95, *p* < 0.001] and Figure 3d [female; *F*_(7,16)_ = 21.65, *p* < 0.001; glucose status main effect: *F*_(1,16)_ = 3.68, *p* = 0.067; treatment main effect: *F*_(3,16)_ = 42.89, *p* < 0.001; glucose status/treatment interaction: *F*_(3,16)_ = 6.39, *p* = 0.002]) expression. Results show that glucose-supplied male astrocytes exhibited reductions in AMPK expression in response to DEX or NE alone; this negative response was exacerbated by DEX plus NE treatment. In the female, AMPK levels were elevated by DEX given alone or together with NE, but this protein was unaffected by NE alone. Glucose withdrawal suppressed AMPK content in male, but not female astrocytes. DEX alone or DEX plus NE decreased or stimulated AMPK expression in male versus female glucose-deprived astrocytes, respectively. Astrocyte pAMPK levels were diminished (male) or augmented (female) by DEX, according to sex. NE stimulated or had no effect on this protein profile in male versus female, respectively. In each sex, pAMPK content was affected similarly by DEX alone versus DEX plus NE treatment. Glucoprivation correspondingly up- (male) or down- (female) regulated pAMPK expression in male and female astrocytes. In glucose-deprived male glial cells, pAMPK profiles were refractory to DEX or NE, but were inhibited by DEX plus NE. Glucose-deprived female astrocytes, on the other hand, showed elevated pAMPK expression in response to all three treatments with the greatest magnitude of increase caused by DEX alone.

**Figure 4:**
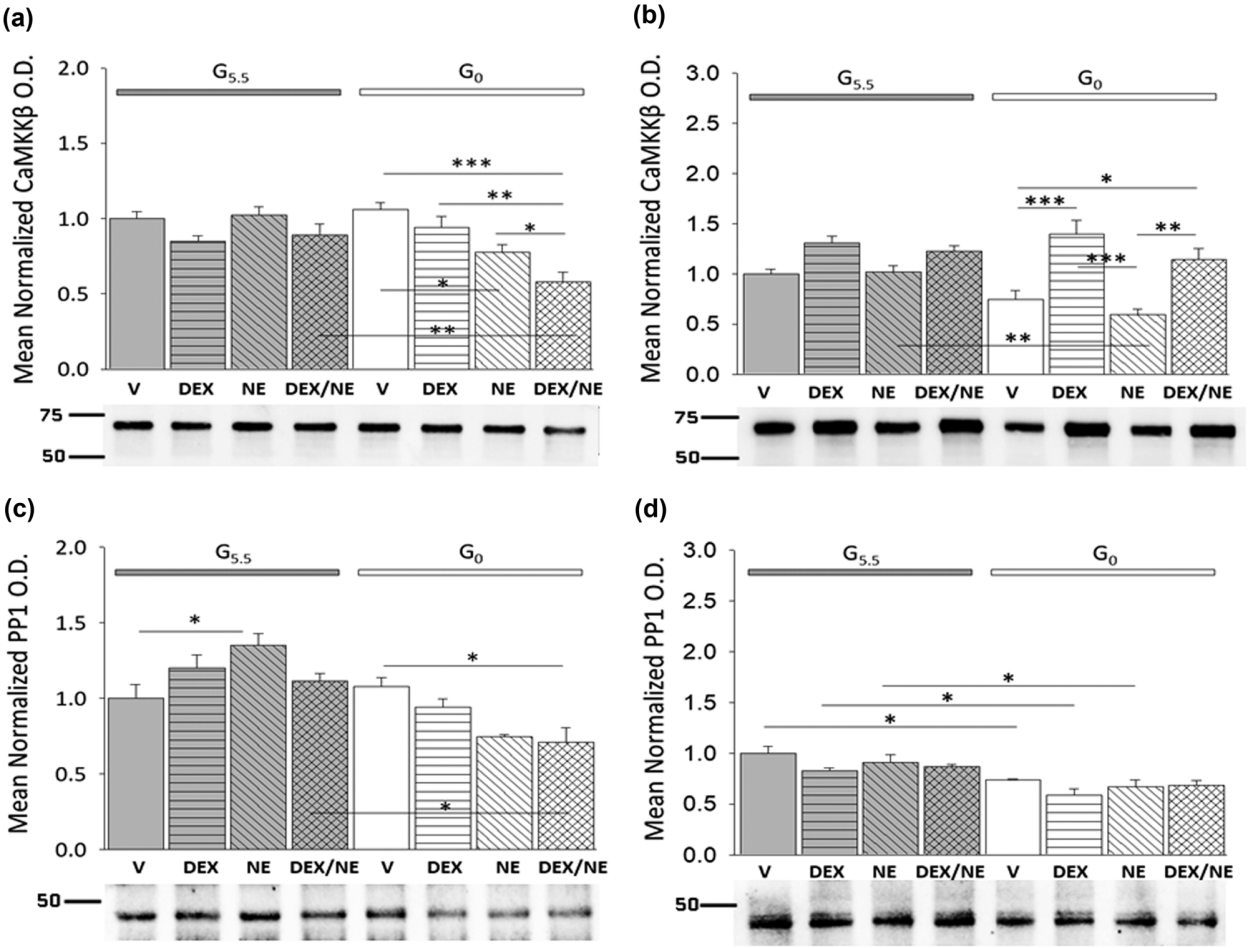
Effects of singular or combinatory DEX and NE treatment on CaMKKβ and PP1 protein profiles in glucose-supplied versus glucose-deprived hypothalamic astrocytes from each sex. Bars depict mean normalized male and female astrocyte CaMKKβ (a: male *n* = 3 independent experiments per treatment group and c: female *n* = 3 independent experiments per treatment group) or PP1 (b: male *n* = 3 independent experiments per treatment group and d: female *n* = 3 independent experiments per treatment group) protein O.D. measures ± S.E.M. after the following treatments: G5.5- or G0-V, G5.5- or G0-DEX, G5.5- or G0-NE, G5.5- or G0-DEX/NE. Data were analyzed by two-way ANOVA and Student Newman Keuls *post-hoc* test. Statistical differences between treatment groups are indicated by the following symbols:*p < 0.05; **p < 0.01; and ***p < 0.001.

AMPK activity is regulated in part by the upstream stimulatory kinase enzyme CaMMKβ as well as the inhibitory phosphatase PP1. It was of interest here to investigate whether action of DEX alone or in coordination with NE controls expression of these enzyme proteins in hypothalamic astrocytes. Figure 4a and b, respectively, depicts DEX and/or NE effects on male (*F*_(7,16)_ = 7.26, *p* = 0.001; glucose status main effect: *F*_(1,16)_ = 6.05, *p* = 0.026; treatment main effect: *F*_(3,16)_ = 8.61, *p* = 0.001; glucose status/treatment interaction: *F*_(3,16)_ = 6.32, *p* = 0.005) or female (*F*_(7,16)_ = 11.13, *p* < 0.001; glucose status main effect: *F*_(1,16)_ = 8.34, *p* = 0.011; treatment main effect: *F*_(3,16)_ = 19.51, *p* < 0.001; glucose status/treatment interaction: *F*_(3,16)_ = 3.67, *p* = 0.035) astrocyte CaMMKβ protein profiles. Data reveal that CaMMKβ expression in glucose-supplied astrocytes of either sex was insensitive to DEX or NE treatment alone or together. NE inhibited this protein in glucose-deprived male cells; this decline was amplified by combinatory NE plus DE treatment. In female astrocytes incubated without glucose, CaMMKβ content was increased by exposure to either DEX or DEX plus NE. Figure 4c and d depicts DEX- or NE-associated patterns of male (*F*_(7,16)_ = 9.63, *p* < 0.001; glucose status main effect: *F*_(1,16)_ = 32.24, *p* < 0.001; treatment main effect: *F*_(3,16)_ = 2.05, *p* = 0.147; glucose status/treatment interaction: *F*_(3,16)_ = 8.34, *p* = 0.005) or female (*F*_(7,16)_ = 6.82, *p* = 0.001; glucose status main effect: *F*_(1,16)_ = 37.94, *p* < 0.001; treatment main effect: *F*_(3,16)_ = 3.09, *p* = 0.057; glucose status/treatment interaction: *F*_(3,16)_ = 0.19, *p* = 0.903) astrocyte PP1 protein levels. NE alone had divergent effects on PP1 expression in male glucose-supplied (increased) versus glucose-deprived (decreased) astrocytes. NE plus DEX had no effect or suppressed PP1 profiles in male astrocytes when glucose was present or absent, respectively. Female astrocyte PP1 content was diminished during glucoprivation, but was insensitive to any drug treatment.

**Figure 5:**
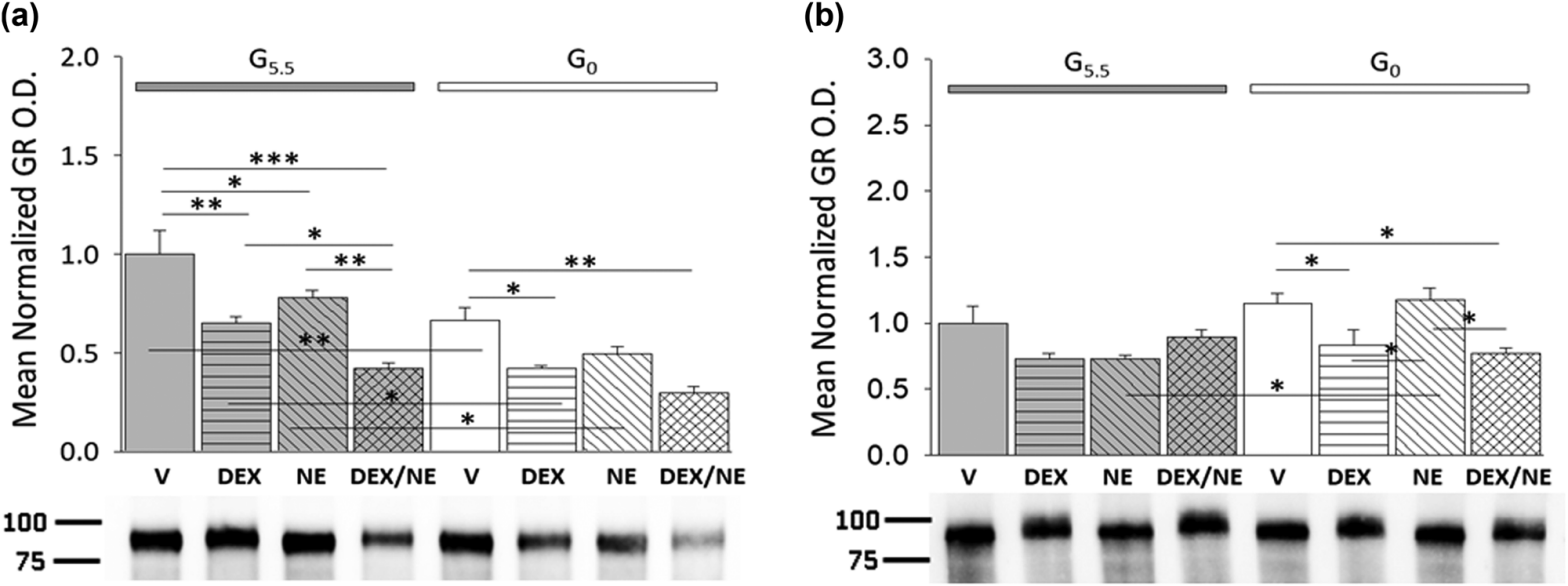
Patterns of hypothalamic astrocyte GR protein expression after treatment with DEX, NE, or DEX plus NE. Data illustrate mean normalized male (a: *n* = 3 independent experiments per treatment group) and female (b: *n* = 3 independent experiments per treatment group) astrocyte GR protein O.D. values ± S.E.M. after the following treatments: G5.5- or G0-V, G5.5- or G0-DEX, G5.5- or G0-NE, G5.5- or G0-DEX/NE. Data were analyzed by two-way ANOVA and Student Newman Keuls *post-hoc* test. Statistical differences between treatment groups are indicated by the following symbols:*p < 0.05; **p < 0.01; and ***p < 0.001.

Research here addressed the issue of whether male and/or female rat hypothalamic astrocyte primary cultures are directly receptive to glucocorticoid control by means of GR expression, and if so, whether this receptor protein profile is sensitive to glucose availability and regulated by glucocorticoid and noradrenergic stimuli in the presence versus absence of glucose. Figure 5 shows effects of DEX and/or NE on male (Figure 5a: *F*_(7,16)_ = 17.04, *p* < 0.001; glucose status main effect: *F*_(1,16)_ = 38.47, *p* < 0.001; treatment main effect: *F*_(3,16)_ = 25.59, *p* < 0.001; glucose status/treatment interaction: *F*_(3,16)_ = 1.35, *p* = 0.295) or female (Figure 5b: *F*_(7,16)_ = 5.14, *p* = 0.001; glucose status main effect: *F*_(1,16)_ = 6.70, *p* = 0.016; treatment main effect: *F*_(3,16)_ = 5.44, *p* = 0.005; glucose status/treatment interaction: *F*_(3,16)_ = 4.31, *p* = 0.014) astrocyte GR protein expression. Data show that GR protein was decreased in G5.5 male astrocytes after incubation with DEX, NE, or DEX plus NE, and that the magnitude of this reduction was greatest in response to the latter combinatory dosing. Meanwhile, GR expression in female G5.5 astrocytes was unaffected by any of these treatments. Glucoprivation did not modify GR expression in astrocytes of either sex. Treatment with either DEX or DEX plus NE resulted in diminished GR protein profiles in G0 male and female astrocytes.

**Figure 6:**
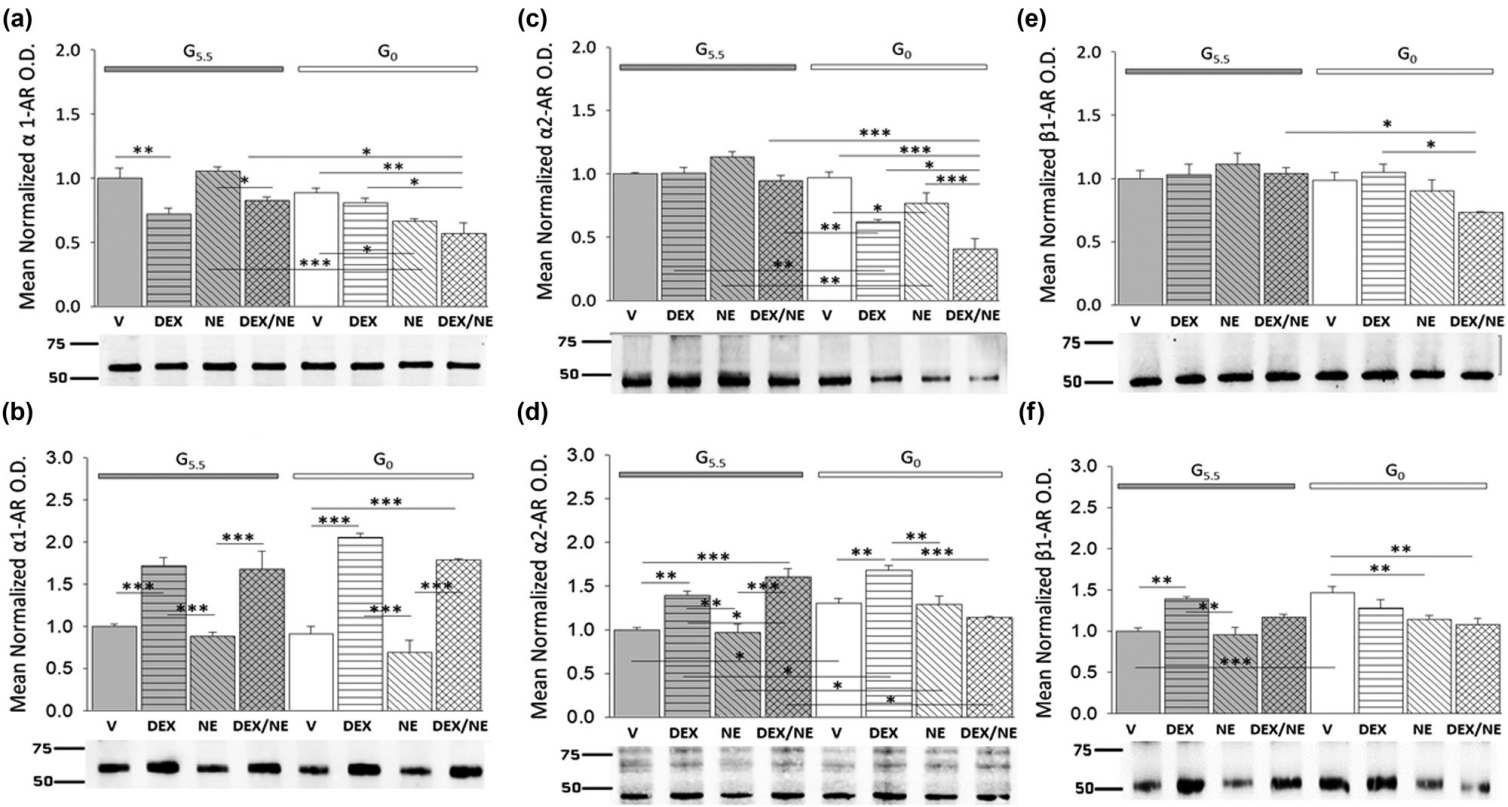
Impact of glucose provision versus starvation on DEX- or NE-associated patterns of α1-AR, α2-AR, and β1-AR protein expression in male and female hypothalamic primary astrocyte cultures. Data illustrate mean normalized α1-AR (a and b: *n* = 3 independent experiments/sex/treatment group), α2-AR (c and d: *n* = 3 independent experiments/sex/treatment group), or β1-AR (e and f: *n* = 3 independent experiments/sex/treatment group) protein O.D. values ± S.E.M. for glucose-supplied (G5.5; gray bars) or glucose-deprived (G0; white bars) male and female astrocytes assigned to the following treatment groups: G5.5- or G0-V (solid bars), G5.5- or G0-DEX (horizontal-striped bars), G5.5- or G0-NE (diagonal-striped bars), G5.5- or G0-DEX/NE (cross-hatched bars). Data were analyzed by two-way ANOVA and Student Newman Keuls *post-hoc* test. Statistical differences between treatment groups are indicated by the following symbols: *p < 0.05; **p < 0.01; and ***p < 0.001.

A critical objective of current work was to examine whether glucose status controls AR variant protein expression in male and female hypothalamic astrocytes, and to determine if DEX and NE act alone or synergistically to regulate these proteins during glucose availability versus deprivation. Figure 6 depicts effects of DEX, NE, or DEX plus NE on hypothalamic astrocyte α1-AR (Figure 6a [male; *F*_(7,16)_ = 10.83, *p* < 0.001; glucose status main effect: *F*_(1,16)_ = 22.84, *p* < 0.001; treatment main effect: *F*_(3,16)_ = 9.43, *p* = 0.001; glucose status/treatment interaction: *F*_(3,16)_ = 8.23, *p* = 0.002] and Figure 6b [female; *F*_(7,16)_ = 23.78, *p* < 0.001; glucose status main effect: *F*_(1,16)_ = 0.27, *p* = 0.608; treatment main effect: *F*_(3,16)_ = 52.99, *p* = <0.001; glucose status/treatment interaction: *F*_(3,16)_ = 2.40, *p* = 0.106]), α2-AR (Figure 6c [male; *F*_(7,16)_ = 20.78, *p* < 0.001; glucose status main effect: *F*_(1,16)_ = 77.61, *p* < 0.001; treatment main effect: *F*_(3,16)_ = 14.40, *p* < 0.001; glucose status/treatment interaction: *F*_(3,16)_ = 8.23, *p* = 0.002] and Figure 6d [female; *F*_(7,16)_ = 14.31, *p* < 0.001; glucose status main effect: *F*_(1,16)_ = 5.52, *p* = 0.032; treatment main effect: *F*_(3,16)_ = 15.74, *p* < .001; glucose status/treatment interaction: *F*_(3,16)_ = 15.80, *p* < 0.001]), and β1-AR (Figure 6e [male; *F*_(7,16)_ = 3.09, *p* = 0.029; glucose status main effect: *F*_(1,16)_ = 7.39, *p* = 0.015; treatment main effect: *F*_(3,16)_ = 2.02, *p* = 0.152; glucose status/treatment interaction: *F*_(3,16)_ = 2.74, *p* = 0.077] and Figure 6 [female; *F*_(7,16)_ = 7.45, *p* < 0.001; glucose status main effect: *F*_(1,16)_ = 5.86, *p* = 0.023; treatment main effect: *F*_(3,16)_ = 7.13, *p* = 0.001; glucose status/treatment interaction: *F*_(3,16)_ = 8.36, *p* = 0.001]) protein expression. Data show that DEX alone inhibited α1-AR protein, but did not regulate α2-AR or β1-AR levels in glucose-supplied male astrocytes. However, DEX alone stimulated each of these AR variant proteins in the female. Glucoprivation did not alter α1-AR, α2-AR, or β1-AR expression profiles in the male, but up-regulated α2-AR and β1-AR levels in female astrocytes. In glucose-deprived male astrocytes, DEX decreased α2-AR content, while NE suppressed α1-AR and α2-AR proteins. DEX plus NE had an equivalent effect to DEX alone on α1-AR expression, but had greater inhibitory effects on α2-AR levels compared to DEX or NE alone. Female glucose-deprived astrocytes showed augmentation of α1-AR and α2-AR, but not by β1-AR by DEX. DEX plus NE increased α1-AR levels to an extent similar to DEX alone, but did not alter α2-AR expression. Both NE alone and NE plus DEX inhibited β1-AR profiles in female astrocytes deprived of glucose.

## 4 Discussion

The current project used a hypothalamic astrocyte primary culture model to address the premise that these glia express glucoprivic-responsive glucose and energy sensing molecular biomarkers, and that glucose-sensitive hormone (glucocorticoid) and/or neurotransmitter (noradrenergic) stimuli govern sensor reactivity to glucose deficiency in a sex-specific manner. Outcomes show that in astrocytes of each sex, membrane glucose sensor GLUT2 and glycolytic pathway sensor GCK protein profiles are controlled by GR and AR input or by glucose, respectively. Yet, expression and activity of the energy sensor AMPK in these cells is subject to sex-dimorphic regulation by DEX, NE, and glucose. Results provide novel evidence that glucose modulates noradrenergic control of astrocyte GLUT2 (both sexes), AMPK (male), pAMPK (both sexes), and α1- and α2-AR (male) proteins. Evidence that co-administration of NE and DEX to male, but not female astrocytes attenuates (GLUT2) or exacerbates (AMPK, GR, β1-AR), effects of DEX alone infers that intersection of GR and AR signaling may be sex-specific. Ongoing work seeks to elucidate mechanisms that underlie differential male versus female astrocyte responses to GR or NE stimulation. There is also a need for understanding of functional implications of glucocorticoid and noradrenergic regulation of hypothalamic astrocyte metabolic sensory functions *in vivo* regarding neural control of glucose homeostasis in each sex.

Present outcomes document expression of the characterized glucose sensors GLUT2 and GCK by hypothalamic astrocytes, and show that GR and AR input regulates the former, but not the latter protein in each sex. Evidence here that DEX treatment augments GLUT2 profiles in astrocytes of either sex, despite dissimilar regulatory effects of DEX on male (decreased) versus female (unchanged) astrocyte GR expression, infers that glucocorticoids may control GR input volume as well as post-receptor signaling in a sex-specific manner. GLUT2 ostensibly employs distinctive structural features to perform glucose transport versus monitoring functions [40]. Data here show that hypothalamic astrocyte GLUT2 expression is unresponsive to glucoprivation. As this protein exhibits low affinity for transportable glucose, constancy of GLUT2 expression over a wide range of ambient tissue glucose levels conceivably allows discriminative, proportionate glucose uptake corresponding to variable extracellular glucose concentrations. It is unclear if GLUT2 detection of extracellular glucose operates with uniform or variable sensitivity over the range of brain tissue glucose concentrations associated with normo- or hypoglycemia. It would be informative to learn if and how GR- or NE-associated up-regulated GLUT2 protein expression impacts glucose reporting and trafficking by this molecule. GLUT2 and GCK are believed to function as tandem elements of a singular glucose-sensing unit in pancreatic beta cells [6], but the prospect that these proteins interact to monitor hypothalamic astrocyte glucose status has not been examined. Our evidence that astrocyte GLUT2 and GCK proteins exhibit disparate responses to distinctive pharmacological and metabolic stimuli infers that these molecules may serve as unique substrates for specific inputs that regulate astrocyte glucose sensing. Notably, co-administration of DEX and NE abolished stimulatory effects of either drug alone on GLUT2 levels in male, but not female, implying that GR and AR signal interaction is sex-specific. Glucoprivic-associated elimination of noradrenergic stimulation of GLUT2 expression supports the presumption that AR regulation of this protein is glucose-dependent in each sex. Although glucose deprivation did not prevent DEX stimulation of GLUT2 protein, the possibility that this metabolic stimulus may affect post-receptor signaling cannot be discounted.

The characterized DEX dosage used here down-regulated astrocyte AMPK and pAMPK in male, but stimulated these proteins in the female, revealing bi-directional, sex-specific glucocorticoid regulation of AMPK expression and activity state in this brain cell type. This divergent hormone action may involve, in part, dissimilar GR signal volume in the two sexes as GR protein expression declined in DEX-treated male astrocytes, but was unaffected by this treatment in the female. DEX may also impose sex-contingent control of downstream post-receptor signaling, transcriptional, and/or post-translational events that govern astrocyte AMPK and pAMPK protein profiles. As the current study focused on a single DEX dose, there would be benefit from future investigation of whether varying GR ligand concentrations elicit proportionate modifications in AMPK and pAMPK levels over all or a segment of a comprehensive, physiological-like dosage range. There also remains a need to assess whether suppressive versus amplifying stimulatory effects of DEX on AMPK and pAMPK expression are associated with specific hormone concentration(s). Our findings that glucoprivation attenuates DEX-induced augmentation of male astrocyte pAMPK content infer that metabolic modulation of GR-mediated action on AMPK activation state may occur in this sex only. Noradrenergic control of this astrocyte energy sensor is evidently also sex-specific as NE incubation suppressed total AMPK protein and stimulated pAMPK levels in male astrocytes, yet had no effect either on protein profile in the female. Notably, glucoprivation was observed to elicit a directional shift in NE control (e.g., negative to positive) on male astrocyte AMPK, and to promote a loss or gain of NE regulation of pAMPK profiles in male versus female, respectively. These data point to sex-dependent metabolic modulation of noradrenergic governance of hypothalamic astrocyte AMPK activity.

Current results show that neither DEX nor NE regulates the upstream stimulatory kinase CaMKKβ in the presence of glucose, yet during glucoprivation DEX elevates this protein profile in the female, while NE suppresses CaMKKβ expression in the male. PP1 levels in glucose-supplied astrocytes of either sex are similarly refractory to DEX, whereas NE enhances this protein in the male only. Notably, the direction of noradrenergic control of PP1 expression in male astrocytes is altered, e.g., switches from stimulatory to inhibitory when glucose is withdrawn. These outcomes support the novel concept that astrocyte energy status may determine whether glucocorticoid and noradrenergic signals are able to regulate astrocyte AMPK activity via upstream enzymes that govern phosphorylation, e.g., during glucose deficiency. DEX treatment of glucose-deprived female astrocytes stimulated CaMKKβ expression without affecting PP1 profiles, coinciding with up-regulated pAMPK. Thus, in this sex, CaMKKβ is likely required for glucocorticoid augmentation of AMPK activity. Interestingly, NE did not modify either CaMKKβ or PP1 expression, but also increased pAMPK profiles, apparently by kinase/phosphatase-independent mechanisms. Glucose-deprived male astrocyte CaMKKβ and PP1 profiles were resistant or inhibited by DEX, respectively, yet pAMPK expression was unaffected by either stimulus, inferring that potential augmentation of pAMPK levels by noradrenergic diminution of PP1 may be counter-balanced by signals that blunt AMPK activation.

It is intriguing to observe that glucoprivic-associated diminution of astrocyte GCK expression coincided with up- or down-regulated pAMPK protein in male versus female, respectively. These findings infer that in the latter sex, augmenting effects of diminished glucose catabolism on sensor activity are apparently offset by regulatory signals that attenuate AMPK phosphorylation and/or decreased allosteric activation of AMPK due to energy production from non-glucose substrates.

Present data also show that NE did not alter any astrocyte AR variant protein profile in either sex, but α1-AR (both sexes), α2-AR (female), and β1-AR (female) proteins are responsive to DEX. The observed efficacy of DEX to up-regulate all three AR variant proteins in the female may contribute to unique effects of combinatory DEX plus NE treatment on specific astrocyte target proteins in that sex. Glucose stability evidently modulates DEX regulation of specific AR proteins in each sex, as glucoprivation abolishes DEX inhibition of α1-AR profiles and allows DEX to suppress α2-AR levels in the male, and in the female eliminates DEX stimulation of β1-AR expression. Glucoprivic governance of noradrenergic regulation of AR protein profiles is, on the other hand, sex-specific as this metabolic stress allows inhibitory noradrenergic control of α1-AR and α2-AR proteins in male hypothalamic astrocytes only.

**Figure 7:**
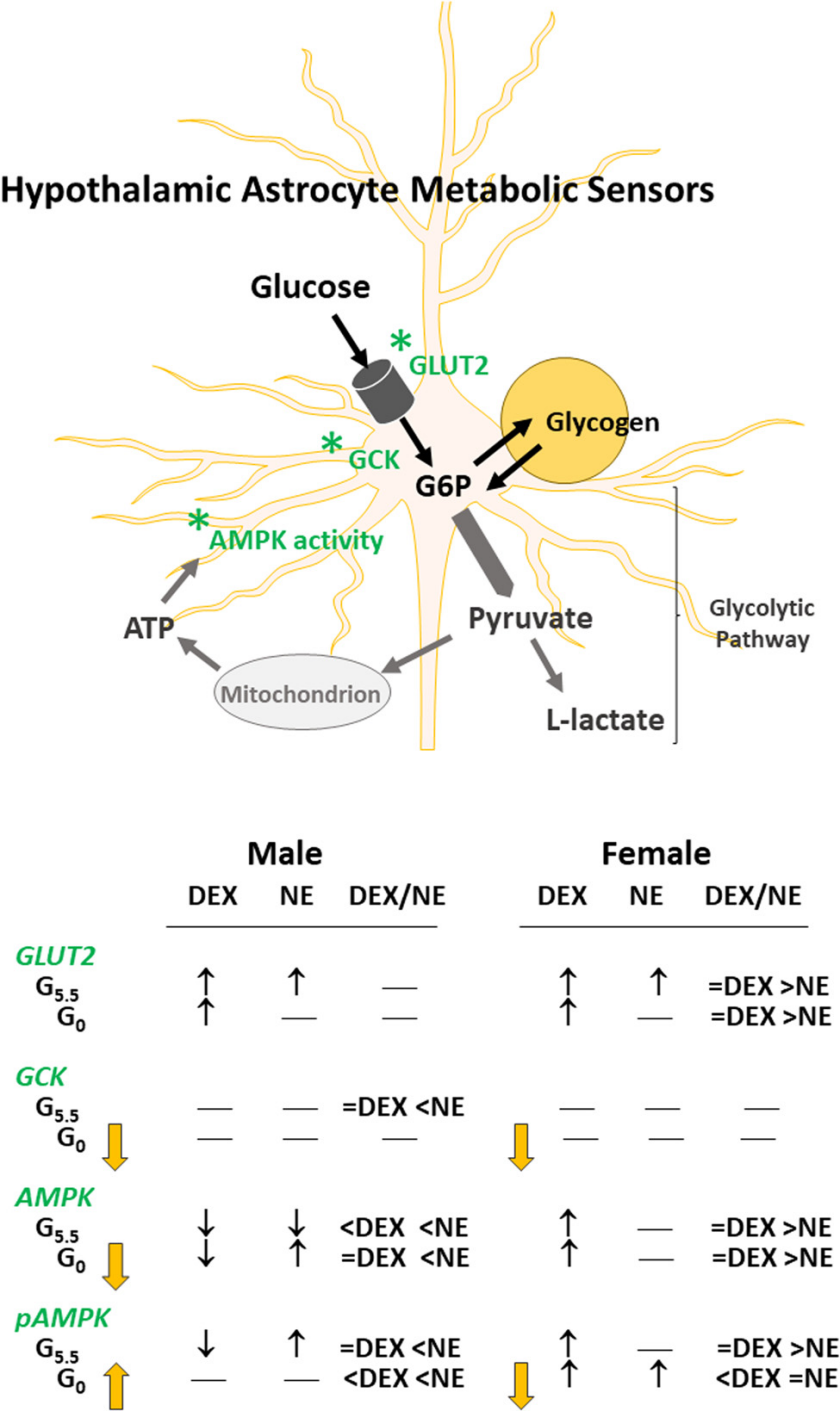
Schematic depiction of sex-specific glucocorticoid and noradrenergic regulation of glucoprivic-sensitive hypothalamic astrocyte nutrient/energy proteins. *Abbreviations:* AMPK: 5′-AMP-activated protein kinase; ATP: adenosine triphosphate; DEX: dexamethasone; GLUT2: glucose transporter-2; G6P: glucose-6-phosphate; GCK: glucokinase (hexokinase IV); NE: norepinephrine; pAMPK: phosphoAMPK. *Symbols*: *: nutrient/energy sensor; 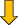 decrease relative to G5.5; ↑: increase versus non-drug-treated controls; ↓: decrease versus non-drug-treated controls; —: no change versus non-drug-treated controls. Summarized here are glucocorticoid and noradrenergic stimulus effects on glucoprivic-sensitive (GCK, AMPK) versus -insensitive (GLUT2) metabolic sensory proteins in each sex. Study outcomes show that DEX and NE impose glucose-dependent control of astrocyte total and pAMPK protein profiles that varies by sex. NE control of specific astrocyte target proteins is also glucose-sensitive, and interaction with GR signaling may be operative in one, but not both sexes.

In summary, current research presents unique proof that hypothalamic astrocytes express membrane and glycolytic pathway glucose-sensory biomarkers in addition to the energy sensor AMPK (Figure 7). Results distinguish glucoprivic-sensitive (GCK, AMPK) versus -insensitive (GLUT2) metabolic sensory proteins, and document regulatory effects of GR or AR signaling on these protein profiles in the presence versus absence of glucose. Current data provide novel evidence for metabolic modulation of NE control of distinctive astrocyte target proteins. Outcomes importantly identify astrocyte AMPK as a target of sex-dimorphic glucocorticoid and noradrenergic control. Observations that co-incubation of male, but not female astrocytes with NE and DEX modifies effects of glucocorticoid signaling alone supports the interaction of GR and AR signaling in one, but not both sexes. Further effort is required to elucidate mechanisms that underlie disparate male versus female astrocyte responses to GR or NE stimulation. There is also a need for research incorporating relevant *in vivo* models of glucose-sensitive glucocorticoid and noradrenergic signaling to the hypothalamus to understand, for each sex, hormonal and neurotransmitter regulation of astrocyte sensing and communication of cellular energetic stability or imbalance.
